# Age at menarche and its implications for the mood-psychosis continuum

**DOI:** 10.1192/j.eurpsy.2025.1076

**Published:** 2025-08-26

**Authors:** M. Puric, M. Nesic, M. Gostiljac, S. Dodic, I. Minic, B. Velickovic, B. Dunjic Kostic, M. Ivkovic, M. Pantovic Stefanovic

**Affiliations:** 1 Department for Bipolar Disorders, Clinic of Psychiatry, University Clinical Centre of Serbia; 2 University of Belgrade, School of Medicine; 3 Department of Organic Mental Disorders, Clinic of Psychiatry, University Clinical Centre of Serbia; 4 Department for Forensic Psychiatry, Institute of Mental Health, Belgrade, Serbia

## Abstract

**Introduction:**

Earlier age at menarche has been associated with an increased risk of affective disorders, but also a later onset of schizophrenia in women, indicating a complex relationship between hormonal changes and mental health. This interplay highlights the importance of understanding how pubertal timing and estrogen exposure can influence both mood disorders and psychotic conditions throughout a person’s life.

**Objectives:**

Our study aimed to explore the relationship between age at menarche and severity of clinical presentation of patients pertaining to mood-psychosis continuum.

**Methods:**

The study group consisted of a total of 109 female patients, 71 diagnosed with bipolar disorder and 38 diagnosed with schizophrenia. The dimensional assessment of psychosis and affective symptoms was done using the Schizo-Bipolar Scale (SBS), together with the severity of the symptom domains measured by the Brief Psychiatric Rating Scale (BPRS).

**Results:**

Age at menarche significantly correlated to score on SBS scale (r= 0.257,p=0.028), after controlling for confounders (age and body mass index). Other clinical characteristics or psychometric properties were not related to age at menarche in the group of BD and SCH patients perceived as a unified group, possibly pertaining to one continuum.

**Conclusions:**

Pubertal timing may play a role in the severity of symptoms associated with bipolar disorder and schizophrenia. Further research is needed to elucidate the specific pathways linking age at menarche to symptom severity and to explore the potential implications for early intervention and treatment strategies targeting the interplay between hormonal factors and mental health outcomes in individuals with bipolar disorder and schizophrenia.

**Disclosure of Interest:**

None Declared

